# Physical Activity and Risk of Cardiovascular Disease—A Meta-Analysis of Prospective Cohort Studies

**DOI:** 10.3390/ijerph9020391

**Published:** 2012-01-26

**Authors:** Jian Li, Johannes Siegrist

**Affiliations:** 1 Mannheim Institute of Public Health, Social and Preventive Medicine, Mannheim Medical Faculty, Heidelberg University, Ludolf-Krehl Strasse 7-11, 68167 Mannheim, Germany; 2 Department of Medical Sociology, University of Düsseldorf, Universitätsstrasse 1, 40225 Düsseldorf, Germany; Email: siegrist@uni-duesseldorf.de; 3 Institute of Occupational and Social Medicine, University of Düsseldorf, Universitätsstrasse 1, 40225 Düsseldorf, Germany

**Keywords:** physical activity, cardiovascular disease, coronary heart disease, stroke, meta-analysis, epidemiological cohort

## Abstract

In order to update and improve available evidence on associations of physical activity (PA) with cardiovascular disease (CVD) by applying meta-analytic random effects modeling to data from prospective cohort studies, using high quality criteria of study selection, we searched the PubMed database from January 1980 to December 2010 for prospective cohort studies of PA and incident CVD, distinguishing occupational PA and leisure time PA, coronary heart disease (CHD) and stroke, respectively. Inclusion criteria were peer-reviewed English papers with original data, studies with large sample size (n ≥ 1,000) and substantial follow-up (≥5 years), available data on major confounders and on estimates of relative risk (RR) or hazard ratio (HR), with 95% confidence intervals (CI). We included 21 prospective studies in the overall analysis, with a sample size of more than 650,000 adults who were initially free from CVD, and with some 20,000 incident cases documented during follow-up. Among men, RR of overall CVD in the group with the high level of leisure time PA was 0.76 (95% CI 0.70–0.82, *p* < 0.001), compared to the reference group with low leisure time PA, with obvious dose-response relationship. A similar effect was observed among women (RR = 0.73, 95% CI 0.68–0.78, *p* < 0.001). A strong protective effect of occupational PA was observed for moderate level in both men (RR = 0.89, 95% CI 0.82–0.97, *p* = 0.008) and women (RR = 0.83, 95% CI 0.67–1.03, *p* = 0.089). No publication bias was observed. Our findings suggest that high level of leisure time PA and moderate level of occupational PA have a beneficial effect on cardiovascular health by reducing the overall risk of incident coronary heart disease and stroke among men and women by 20 to 30 percent and 10 to 20 percent, respectively. This evidence from high quality studies supports efforts of primary and secondary prevention of CVD in economically advanced as well as in rapidly developing countries.

## 1. Introduction

Cardiovascular disease (CVD), including coronary heart disease (CHD) and stroke, is a major contributor to the World’s burden of disease, ranging currently as the most important cause of death and producing substantial disability and reduced well-being among surviving people [1,2]. Distinct measures of primary prevention, mainly regular physical activity (PA), healthy diet, and smoking cessation have been investigated, with convincing evidence of respective risk reductions of cardiovascular morbidity and mortality [3,4]. This evidence includes studies of protective effects of regular PA against CHD, but fewer—and less consistent—investigations were conducted with regard to protective effects against stroke [5,6]. In addition, most of early reports on the relationship between PA and CVD were focused on men, and the few studies exploring associations of PA with CVD among women demonstrated conflicting results, in part due to the fact that different types of activity, their duration and intensity were monitored [7,8]. More importantly, the majority of existing evidence of health benefits relates to leisure time PA whereas another principal type of physical activity, *i.e.*, occupational PA has been less thoroughly investigated [9]. Both leisure time and occupational PA are generally considered to provide protective effects on health, however, recent studies observed that heavy occupational PA might be harmful to health [10]. Another crucial question is how much PA is good for cardiovascular health. It is still unclear whether more PA is even better, as expected from research on dose-response relationships [11].

In view of these inconsistencies an updated systematic review of the current state of knowledge seems warranted, and we set out to contribute to this aim by reviewing available evidence from prospective epidemiological cohort studies. In epidemiological research the prospective observational study is considered a gold standard approach because of its temporal sequence (exposure assessment precedes disease onset), its sample size (based on statistical power calculation and allowing for adjustment for confounding variables in multivariate analysis), and the quantification of subsequent disease risk following exposure (relative risk of disease in exposed *versus* non-exposed individuals). Prospective cohort studies are expensive and time-consuming, but due to their methodological strengths their findings provide a strong case of credibility, in particular if supported by results from intervention studies demonstrating CVD risk reduction as a function of increased PA. We therefore performed this systematic review with an exclusive focus on findings from prospective cohort studies of different types of PA (occupational and leisure time PA) and different types of CVD (CHD and stroke), with special emphasis on a potential dose-response relationship. Importantly, where possible we separated the findings among men from those among women to explore potential gender-specific associations. Results are expected to strengthen the evidence base of measures of primary and secondary prevention of cardiovascular risks and diseases.

## 2. Methods

### 2.1. Study Selection and Data Extraction

We applied the internationally established quality criteria for performing systematic review and meta-analysis of epidemiological studies [12,13]. The following inclusion criteria were applied to the process of literature search, in an effort to identify most convincing evidence: (1) Database: PubMed; (2) Time duration: January 1980–December 2010; (3) Medical subject headings and terms: physical activity, exercise, cardiovascular disease, coronary heart disease, stroke, cerebrovascular disease; (4) Study design: prospective cohort studies; (5) Peer-reviewed English articles with original data; (6) Healthy populations without history of disease of interest; (7) Sample size: ≥1,000; (8) Follow-up: ≥5 years; (9) Adjustment for relevant confounding factors; (10) Estimation of relative risk (RR), or hazard ratio (HR), with 95% confidence interval (CI); (11) Objective measurement of PA; (12) At least 3 or more categories of occupational or leisure time PA. Information on study design, participant characteristics, measurement of exposure and outcome variables, adjustment for potential confounding, and estimates of associations was abstracted.

### 2.2. Statistical Analysis

The random effects modeling approach was used to perform meta-analysis [14]. We distinguished three levels of PA: high, moderate, and low. The lowest category was defined as low level PA (reference group), the highest category as high level PA. All categories in between were pooled to represent a moderate level PA [5]. For each selected study we extracted a RR or HR for the high *versus* the low PA group, and for the moderate *versus* the low PA group, respectively. These separate analyses can be interpreted as an estimate of a potential dose-response relationship between levels of occupational/leisure time PA and risk for CHD/stroke. We used the Q-test for heterogeneity of study results. To detect publication biases we explored heterogeneity in funnel plots and the degree of asymmetry by using Begg’s asymmetry method [15]. Data for CHD and stroke were analyzed separately in a first step, followed by a step where these measures were combined. In all analyses, conducted by the statistical program STATA 11, occupational and leisure time PA, men and women were analyzed separately [16].

## 3. Results

The systematic search identified 1974 potentially relevant articles according to the inclusion criteria (1), (2), and (3). Of these, 31 articles met the inclusion criteria (4), (5), (6), (7), (8), (9), and (10). Furthermore, 10 articles were excluded based on the inclusion criteria (11) and (12). Then 21 papers included the following endpoints: 12 studies with fatal or non-fatal manifestation of CHD [17,18,19,20,21,22,23,24,25,26,27,28], and nine studies with fatal or non-fatal stroke [29,30,31,32,33,34,35,36,37] (see Table 1). Eight studies were conducted exclusively among men, and seven studies concerned women, whereas the remaining papers included men and women. A majority of 16 studies were conducted in the USA, and five studies originated from Europe (three from Finland and two from UK). All studies investigated leisure time PA whereas three studies involved separate measurements of occupational PA. The population of this meta-analysis included more than 650,000 adults who were initially free from CHD or stroke, and some 20,000 incident cases were documented during follow-up.

**Figure 1:**
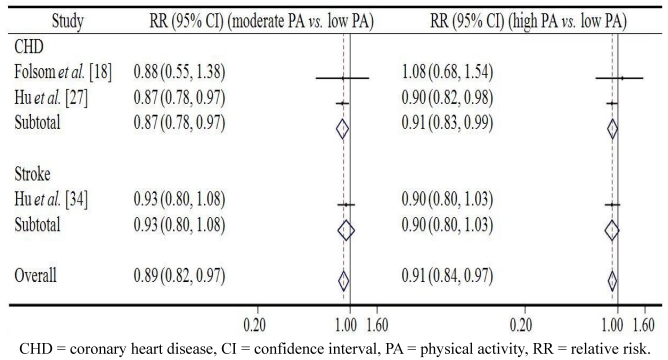
The association between occupational physical activity and cardiovascular disease from prospective cohort studies in men.

**Figure 2:**
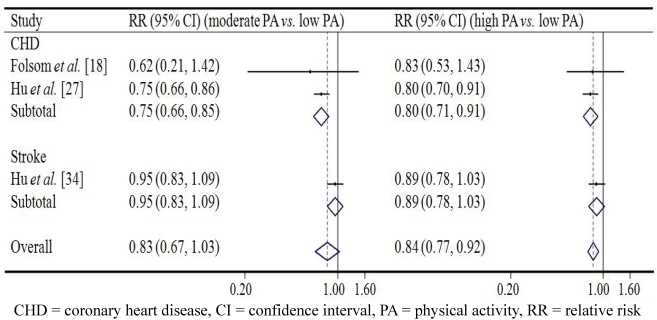
The association between occupational physical activity and cardiovascular disease from prospective cohort studies in women.

Figure 1 and Figure 2 demonstrate the associations between occupational PA and CVD in men and in women respectively. Among men, the pooled relative risk (RR) of overall CVD in the group with the moderate level of occupational PA was 0.89 (95% CI 0.82–0.97, *p* = 0.008) compared to the reference group with low occupational PA; while high level of occupational PA seemed not have more protective effect on overall CVD (RR = 0.91, 95% CI 0.84–0.97, *p* = 0.006). A similar effect was observed in case of both CHD and stroke: moderate occupational PA predicted lowest risk of CHD and stroke. Among women, results were in line with those reported for men. The pooled RR of overall CVD in the group with moderate level of occupational PA was 0.83 (95% CI 0.67–1.03, *p* = 0.089) compared with the reference group with low occupational PA, while high level of occupational PA was associated with a reduced risk of overall CVD (RR = 0.84, 95% CI 0.77–0.92, *p* < 0.001). Again, similar effects for CHD and stroke were observed.

**Figure 3:**
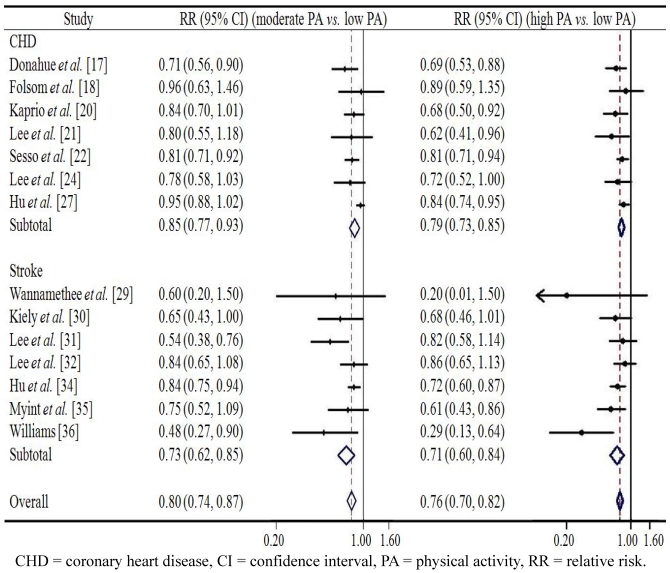
The association between leisure time physical activity and cardiovascular disease from prospective cohort studies in men.

**Figure 4:**
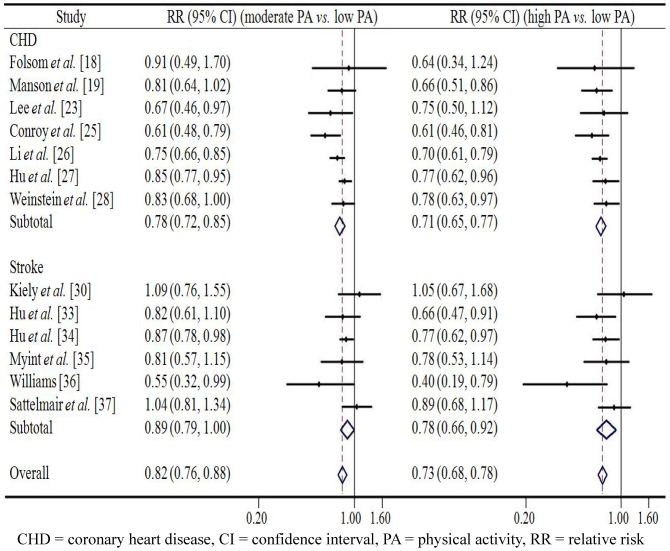
The association between leisure time physical activity and cardiovascular disease from prospective cohort studies in women.

Figure 3 and Figure 4 present the associations between leisure time PA and CVD in men and in women respectively. Among men, the pooled RR of overall CVD in the group with the moderate level of leisure time PA was 0.80 (95% CI 0.74–0.87, *p* < 0.001) compared to the reference group with low leisure time PA; while a high level of leisure time PA showed a somewhat stronger protective effect against overall CVD (RR = 0.76, 95% CI 0.70–0.82, *p* < 0.001). A similar effect was observed in case of both CHD and stroke: high leisure time PA was associated with lowest risk of CHD (RR = 0.79) and stroke (RR = 0.71) respectively. Among women, similar pattern was found with men. The pooled RR of overall CVD in the group with moderate level of leisure time PA was 0.82 (95% CI 0.67–0.88, *p* < 0.001) compared with the reference group with low leisure time PA, while a high level of leisure time PA reduced the risk of overall CVD to 0.73 (95% CI 0.68–0.78, *p* < 0.001). Similarly, the lowest risks of CHD (RR = 0.71) and stroke (RR = 0.78) were found in people with high level of leisure time PA. Despite the overall significantly reduced relative risks of CVD among men and women who were physically active to a substantial extent the confidence intervals in six or seven studies reached beyond the margin of 1.0, thus indicating a non-significant effect. We found no evidence of publication bias in any analyses using Begg’s asymmetry method (*p* > 0.10).

**Table 1:** Summary of prospective cohort studies on physical activity and cardiovascular disease.

Study	Country	Population	Follow-up duration	Measurement of physical activity	No. of cases	Adjustments
CHD						
Donahue *et al.* [17]	USA	7,644 men, age 45–69 years	12 years	Leisure time PA index based on average time of activities	888 cases	Age, alcohol, smoking
Folsom *et al.* [18]	USA	6,188 men and 7,852 women, age 45–64 years	Up to 7 years	Leisure time PA and occupational PA indices based on average frequency of activities	320 cases	Age, race, study center, education, smoking, alcohol, hormone replacement therapy (women), diabetes, waist/hip ratio, cholesterol, blood pressure, anti-hypertensive medication use, fibrinogen.
Manson *et al.* [19]	USA	72,488 women, age 40–65 years	8 years	Leisure time PA score based on average time of activities, and expressed as energy expenditure	645 cases	Age, period during the study, smoking, BMI, menopausal status, parental history of myocardial infarction, vitamin-supplement, alcohol, history of hypertension, diabetes, hypercholesterolemia, and aspirin use
Kaprio *et al.* [20]	Finland	8,205 twin men, age 25–69 years	Up to 20 years	Leisure time PA score based on average frequency, duration, and intensity of activities, and expressed as energy expenditure	723 cases	Age, BMI, smoking, hypertension, diabetes
Lee *et al.* [21]	USA	7,307 men, mean age 66.1 years	5 years	Leisure time PA score based on average frequency and duration of activities, and expressed as energy expenditure	482 cases	Age, duration per exercise episode, smoking, hypertension, diabetes, early parental death, vitamin/mineral supplements, alcohol, red meat and vegetable consumption, participation in vigorous activities
Sesso *et al.* [22]	USA	12,516 men, mean age 57.7 years	Up to 16 years	Leisure time PA score based on average frequency and duration of activities, and expressed as energy expenditure	2,135 cases	Age, BMI, alcohol, hypertension, diabetes, smoking, early parental death
Lee *et al.* [23]	USA	39,372 healthy women, age 45 years or older	Average 5 years	Leisure time PA score based on average time of activities, and expressed as energy expenditure	244 cases	Age, smoking, alcohol, diet, menopausal status, use of postmenopausal hormones, and parental history of early myocardial infarction
Lee *et al.* [24]	USA	7,337 men, mean age 66 years	Average 5.3 years	Leisure time PA score based on average frequency, duration, and intensity of activities, and expressed as energy expenditure	551 cases	Age, smoking; alcohol, red meat, vegetables, early parental mortality, BMI, history of hypertension, cholesterol, and diabetes
Conroy *et al.* [25]	USA	37,169 healthy women, age 45 years or older	Average 9 years	Leisure time PA score based on average time of activities, and expressed as energy expenditure	477 cases	Age, smoking, alcohol, diet, use of hormone therapy, menopausal status, and family history of CHD
Li *et al.* [26]	USA	88,393 women, age 34–59 years	20 years	Leisure time PA index based on average time of activities	2,358 cases	Age, smoking, parental history of CHD, postmenopausal status and hormone use, aspirin use, alcohol consumption, and BMI
Hu *et al.* [27]	Finland	47,840 men and women, age 25–64 years	18.9 years	Leisure time PA and occupational PA indices based on average intensity of activities	4,660 case	Age, BMI, blood pressure, cholesterol, education, alcohol, smoking, history of diabetes
Weinstein *et al.* [28]	USA	38,987 women, age 45 years or older	10.9 years	Leisure time PA score based on average time of activities, and expressed as energy expenditure	948 cases	Age, parental history of myocardial infarction, alcohol, smoking, use of hormone therapy, and dietary factors
Stroke						
Wannamethee *et al.* [29]	UK	5,694 men, age 40–59 years	Up to 9.5 years	Leisure time PA score based on average frequency, duration, and intensity of activities, and expressed as energy expenditure	68 cases	Age, smoking, BMI, social class, drinking
Kiely *et al.* [30]	USA	3,258 men and 4,161 women, mean age 55 years	Up to 32 years	Leisure time PA index based on average time of activities	680 cases	Age, occupation, blood pressure, smoking, cholesterol, total vital capacity, BMI, glucose intolerance, atrial fibrillation, left ventricular hypertrophy, valvular disease, history of congestive heart failure and ischemic heart disease
Lee *et al.* [31]	USA	11,130 men, mean age 58 years	Up to 13 years	Leisure time PA score based on average frequency and duration of activities, and expressed as energy expenditure	378 cases	Age, smoking, alcohol, early parental death
Lee *et al.* [32]	USA	21,823 men, age 40–84 years	11.1 years	Leisure time PA index based on average frequency of vigorous exercise	533 cases	Age, smoking, alcohol, BMI, history of hypertension, high cholesterol, diabetes, angina, parental history of early myocardial infarction
Hu *et al.* [33]	USA	72,488 women, age 40–65 years	8 years	Leisure time PA score based on average time of activities, and expressed as energy expenditure	258 cases	Age, smoking, BMI, menopausal status, parental history of early myocardial infarction, alcohol, aspirin, history of hypertension, diabetes, or hypercholesterolemia
Hu *et al.* [34]	Finland	47,721 men and women, age 25–64 years	19 years	Leisure time PA and occupational PA indices based on average intensity of activities	2,863 case	Age, area, study year, BMI, blood pressure, cholesterol, education, smoking, alcohol, diabetes
Myint *et al.* [35]	UK	22,602 men and women, age 40–79 years	8.6 years	Combined leisure time PA and occupational PA index based on average time and intensity of activities	361 cases	Age, blood pressure, BMI, cholesterol, smoking, history of diabetes
Williams [36]	USA	29,279 men and 12,123 women, mean age 44.8 years for men and 38.9 for women	7.7 years	Running distance	119 cases	Age, smoking
Sattelmair *et al.* [37]	USA	39,315 healthy women, age ≥ 45 years	11.9 years	Leisure time PA score based on average time of activities, and expressed as energy expenditure	579 cases	Age, smoking, alcohol, diet, menopausal status, parental history of myocardial infarction, migraine aura, BMI, history of diabetes, elevated cholesterol, hypertension

BMI = body mass index, CHD = coronary heart disease, PA = physical activity.

## 4. Discussion

This present review examined the association between PA and risk of CVD with a meta-analysis of 21 prospective cohort studies of men and women who were free of CVD at study entry. The overall population size reached beyond 650,000 adult individuals, followed-up over a period ranging from 5 to 32 years. There were more than 20,000 incident cases. The rather consistent results among men and women suggest that high level of leisure time PA reduces the risk of CVD in a range of about 20 to 30 percent, compared to the risk of those with low level of PA at leisure time, while moderate leisure time PA decreases the risk by about 10–20 percent, indicating an obvious dose-response relationship. However, a moderate level of occupational PA is associated with a 10 to 20 percent lower risk of CVD, whereas high PA at work fails to show any stronger protective effect against CVD. These effects are independent of the impact of major cardiovascular risk factors which were considered as confounders.

According to the U.S. 2008 physical activity guidelines, it is recommended that “all adults should avoid inactivity. Some physical activity is better than none, and adults who participate in any amount of activity gain some health benefits” and “additional benefits occur with more physical activity” [38]. The guidelines have been well supported by a recent review, particularly on leisure time PA and CHD [39]. Yet, the evidence of a dose-response relationship between PA and stroke is not as consistent as in case of CHD outcome [5,8,40]. The results from our review, using strict inclusion criteria to maintain high quality studies only, provide some evidence of a dose-response relationship between leisure time PA and stroke. A similar relationship in case of occupational PA and CVD is not yet established.

Although working life witnessed dramatic changes during the past few decades, a considerable proportion of employed population is still exposed to strenuous physically work requiring a high amount of energy expenditure. In developed countries, it is estimated that physical activity at work accounts for about 30 percent of total physical activity [41]. In recent years, several studies questioned whether occupational PA exerts similar protective effect on health (particularly cardiovascular health) similar to the ones observed in leisure time PA. Recent evidence even suggests opposite effects of the two types of PA (occupational and leisure time) on health, such that occupational PA may be harmful to health, whereas leisure time PA exerts beneficial effects on health [10]. This phenomenon is in line with the traditional occupational health view that heavy physical job demand is considered an occupational health hazard [42]. Our current meta-analysis at least partly suggests that, while moderate level of occupational PA reduces the risk of CVD, high PA at work does not add to a potential protective effect. Nevertheless, we have to bear in mind that only three studies with respect to occupational PA were included in our meta-analysis. Therefore, additional epidemiological studies exhibiting a high quality are needed in this case. These studies should also consider the differential time spent with PA between occupational and leisure time activity, as well as differential options of exerting control over, and of expecting psychological benefits from PA [41].

It is of interest to note that the size of effects was well comparable across the different diagnostic groups, *i.e.*, CHD and stroke. Although additional research is required to identify the shared and differential processes mediating PA with CHD and stroke respectively, the following conclusions can be made on the basis of current evidence: First, in case of stroke and of CHD a reduction of shared main risk factors, including reduced blood pressure, body weight, decrease in LDL- and increase in HDL cholesterol, and maintenance of normal glucose tolerance contribute to risk reduction, but studies adjusting for these effects indicate a relatively small contribution. Second, additional physiological responses associated with lack of PA that carry potential pathogenic effects on general atherosclerosis (affecting CHD and stroke) concern reduced endothelial function, elevated thrombogeneity, heightened imbalance of autonomic nervous system, increased endogenous inflammation and coagulation factors (VII, IX, vWF). Third, some of the protective factors reinforced by PA may be responsible for risk reduction in CHD specifically, such as attenuated plaque progression in coronary arteries, enhanced collateralization of coronary arteries, or infarct sparing due to myocardial preconditioning [43,44,45]. In addition, there are protective effects resulting from body weight control and its effect on high blood pressure and risk of metabolic syndrome [46].

During the past decade, several reviews and meta-analyses have examined the relationship between PA and CVD [3,5,6,7,8]. However, these meta-analyses evaluated PA and CVD as an overall category, without distinguishing between occupational PA and leisure time PA, and between CHD and stroke. In addition, few explored the dose-response relationship [39,40]. Moreover, several reports focused exclusively on men. These limitations were overcome by the current report that includes different levels of occupational and leisure time PA and two major types of CVD (CHD and stroke) in men and women, respectively. We decided to restrict the analysis to prospective cohort studies with a large sample size and with a relatively long duration of follow-up in order to improve the reliability and validity of findings. While these restrictions resulted in a smaller number of studies included in the meta-analysis compared to some previous reports, we maintain that quality of studies matters more than quantity. In this contribution, we also examined potential publication bias but did not detect any substantial effect.

Several potential limitations have to be addressed. First, as is the case in a majority of epidemiological studies exploring associations of PA with health, it is difficult to elucidate the specific role of PA as part of a health-related lifestyle that produces favorable effects on diet, body weight control and reduction or lack of addictive behaviors, such as cigarette smoking or heavy alcohol consumption. Adjusting for these factors in multivariate statistical analysis has been performed in many of the reported studies, but this approach may not do justice to the complex web of causation produced by comprehensive health-related lifestyles. A second limitation relates to the accurate assessment of PA by questionnaire. Unfortunately, there was no uniform measurement in the 21 studies included. Rather, assessments varied quite substantially with regard to frequency, intensity and duration of PA [47]. In this context, we divided PA into two broad types, *i.e.*, occupational PA and leisure time PA, and included only objective measurement of PA, even though a recent meta-analysis indicates that a significant protective effect of PA on health is not compromised by the fact that self-report data are often less valid than objective measurements [3]. Third, hemorrhagic stroke and ischemic stroke were not analyzed separately in our review. Given the differences in etiology, it might be suspected that PA affects hemorrhagic and ischemic stroke differently. However, previous meta-analyses suggested that the protective effects of PA were similar in hemorrhagic and ischemic stroke [5,6]. Last, all studies included in this meta-analysis originated either from the USA or from Europe. This restriction is in part due to some of the selection criteria (especially papers published in English-language journals), but may also reflect the fact that the burden of disease in economically advanced Western countries with its shift from communicable to non-communicable diseases occurred earlier than in other parts of the world and, thus, raised new public health concerns for research and policy [1,2]. In view of the current socioeconomic and epidemiologic transition in rapidly developing countries, and in particular in many Asian countries, respective evidence on protective effects of CVD produced by regular PA is badly needed [48,49,50].

In conclusion, the results of this meta-analysis suggest that high level of leisure time physical activity has a beneficial effect on cardiovascular health by reducing the overall risk of incident CHD and stroke among men and women by 20 to 30 percent, while moderate level of occupational physical activity might reduce 10 to 20 percent risk of CVD. Results are in line with available evidence from previous reviews, but improved estimates may be due to the high quality of selection criteria of the studies included in this analysis. The findings have obvious direct implications for primary and secondary prevention, not only in Western countries, but equally so in rapidly developing countries in Asia and other regions of the world.
